# QR Code–Enabled Self-Access Lifestyle Education in Older People Living With HIV: Pragmatic Pilot Quasi-Experimental Pretest-Posttest Implementation Study

**DOI:** 10.2196/93633

**Published:** 2026-07-29

**Authors:** Yamal Jamal-Ismail Ortiz, Santiago Vico, Javier Pérez-Stachowski, Jimena Abilés, Pedro Alarcón, Javier Arenas-Villafranca, Francisco Rivas-Ruiz, Alfonso Del Arco, María Dolores Martín-Escalante, Julián Olalla

**Affiliations:** 1 Department of Internal Medicine Hospital Costa del Sol Marbella, MA Spain; 2 Departamento de Biomedicina y Odontología, Facultad de Ciencias Biomédicas y Deporte, Universidad Europea de Andalucía, 29010 Málaga Spain; 3 Department of Physiotherapy Hospital Costa del Sol Marbella, MA Spain; 4 Department of Hospital Pharmacy Hospital Costa del Sol Marbella, MA Spain; 5 Research and Innovation Unit Hospital Costa del Sol Marbella, MA Spain

**Keywords:** HIV, frailty, older adults, QR code enabled, digital health, self-management, Mediterranean diet, exercise, insomnia, loneliness

## Abstract

**Background:**

Frailty is prevalent and dynamic in older people living with HIV and is associated with adverse outcomes. Lifestyle support is recommended but difficult to deliver at scale. Digital self-access education may help, although evidence in older, multimorbid populations is limited.

**Objective:**

This study aims to evaluate 6-month changes in frailty phenotype and related outcomes after a QR code–enabled self-access lifestyle education program on Mediterranean diet and exercise routines for people living with HIV aged ≥60 years.

**Methods:**

We conducted a pragmatic, single-arm, quasi-experimental exploratory pretest-posttest evaluation in the HIV outpatient clinic of University Hospital Costa del Sol (Marbella, Spain). Participants received a trifold leaflet with QR codes linking to curated YouTube videos on Mediterranean diet and aerobic or resistance exercise, as an adjunct to usual care. Frailty was assessed using the Fried frailty phenotype. Secondary outcomes included frailty criteria, patient-reported measures (Insomnia Severity Index [ISI], 10-item Connor-Davidson Resilience Scale [CD-RISC-10], University of California, Los Angeles, Loneliness Scale [ULS], and Hospital Anxiety and Depression Scale categories), physical activity (International Physical Activity Questionnaire), Mediterranean diet adherence (MEDAS), and inflammatory and immunologic markers. Frailty transitions were summarized descriptively; paired dichotomous variables were analyzed with McNemar test, and continuous variables with a paired *t* test or Wilcoxon signed-rank test. All tests were 2-tailed.

**Results:**

Of 52 enrolled participants, 50 (96.2%) were included at 6 months. Frailty transitions were frequent and bidirectional and occurred only between adjacent states. Of 4 frail participants at baseline, 2 transitioned to prefrail; of 25 prefrail participants, 5 improved to robust and 6 progressed to frail; and of 21 robust participants, 10 transitioned to prefrail. No statistically significant changes were observed in inflammatory and immunologic markers, physical activity, or MEDAS scores. Insomnia improved (from a median ISI score of 6.5, IQR 4-9 to a median of 4.0, IQR 2-7; *P*=.001; *r*=0.49), resilience increased (median CD-RISC-10 32.0, IQR 28-36 to 37.0, IQR 33-40; *P*<.001; *r*=0.65), and loneliness worsened (median ULS 34.5, IQR 30-39 to 38.0, IQR 34-42; *P*=.001; *r*=0.49). Grip strength did not improve among participants with impaired baseline strength.

**Conclusions:**

In this uncontrolled exploratory study, selected psychosocial outcomes changed over 6 months after delivery of a QR code–enabled lifestyle education strategy, whereas no clear short-term changes were observed in lifestyle, strength, or biomarker outcomes. Findings should be interpreted as exploratory and hypothesis-generating rather than causal.

## Introduction

The population of people living with HIV is aging, and frailty has emerged as a clinically meaningful syndrome that reflects decreased physiological reserve and increased vulnerability to stressors. Frailty is associated with morbidity, falls, hospitalization, and mortality in older adults, including people living with HIV. The Fried frailty phenotype remains one of the most widely used measures and allows for comparison across cohorts [1,2].

In people living with HIV, frailty is increasingly recognized as a fluctuating condition with frequent transitions between adjacent states over time influenced by multimorbidity, immunologic and inflammatory factors, and psychosocial determinants such as depression and loneliness. Contemporary cohorts have highlighted that loneliness and social vulnerability are associated with frailty and adverse outcomes among older people living with HIV [3-5].

Lifestyle interventions—particularly increasing physical activity (including resistance training) and improving diet quality—are consistently recommended as core components of frailty prevention and mitigation. However, implementation is constrained by staff time, access to supervised programs, and patient barriers (transportation, stigma, and competing comorbidities). Digital tools that enable self-access to educational content may offer scalable support, but adoption in older adults with chronic diseases can be limited by digital literacy, usability, access to devices, and motivational factors [1,6].

Digital tools may offer scalable solutions to support lifestyle interventions in older adults; however, evidence on their effectiveness remains heterogeneous, particularly when interventions lack interactive or socially engaging components [7]. Moreover, adoption of digital health interventions in older populations is often constrained by barriers such as digital literacy, usability, and accessibility, which may limit sustained engagement and impact [6,8].

QR codes represent a low-cost mechanism to bridge printed materials and digital education, enabling rapid access to videos and other resources without requiring installation of dedicated apps. Prior literature suggests generally positive perceptions of QR code–based approaches but also highlights infrastructure and usability barriers, especially in older populations [9,10]. This study aimed to assess 6-month outcomes after delivering a QR code–enabled, self-access lifestyle education program (Mediterranean diet and exercise videos) to people living with HIV aged 60 years and older, focusing on frailty transitions and changes in physical, behavioral, psychosocial, and inflammatory and immunologic measures.

## Methods

### Study Design and Setting

This study was designed as a pragmatic, single-arm exploratory pretest-posttest outcome evaluation of a QR code–enabled, self-access digital lifestyle education strategy implemented under routine care conditions. Although the intervention was implemented pragmatically in clinical practice, this study was not designed as a full implementation evaluation and did not assess core implementation outcomes such as reach, adoption, usability, acceptability, fidelity, or intervention use metrics.

### Participants

Participants were identified from an HIV outpatient clinic registry and contacted via telephone by study investigators to assess eligibility and invite them to take part. Recruitment was conducted consecutively among eligible patients aged 60 years and older with stable residence in the Costa del Sol Health District catchment area. Key exclusions were designed to avoid enrolling individuals with substantial dependency or conditions preventing safe exercise participation, including Barthel index below 90, life expectancy of less than 1 year, Child-Pugh class C for cirrhosis, renal replacement therapy, dementia, recent fracture limiting walking or grip strength, recent major surgery, and active malignancy (with specified exceptions).

### Ethical Considerations

This study was approved by the Research Ethics Committee of Area Costa del Sol (approval date: March 3, 2022; reference number 001_feb22_PI – Fragilidad_VIH). All participants provided written informed consent prior to inclusion. Participant confidentiality was ensured in accordance with the Declaration of Helsinki, the European General Data Protection Regulation (Regulation [EU] 2016/679), and Spanish data protection laws (Organic Law 3/2018 on the Protection of Personal Data and Guarantee of Digital Rights and Law 14/2007 on Biomedical Research). Personal identifiers were stored separately from study data, and all analyses were conducted on anonymized datasets using coded identifiers. Access to identifiable data was restricted to study investigators and the ethics committee. No financial compensation was provided to participants.

### Intervention: QR Code–Enabled, Self-Access Lifestyle Education

At baseline, participants received a trifold leaflet containing QR codes linking to YouTube videos accessible via personal electronic devices (smartphone and tablet; Multimedia Appendix 1). The QR-linked materials consisted of short videos (10-15 minutes) focused on maintaining a balanced Mediterranean diet developed by our hospital’s nutrition department, as well as on initiating and adhering to a progressive aerobic and anaerobic exercise program developed by our hospital’s physiotherapy department. The videos provided Mediterranean diet education and practical guidance, aerobic and cardiovascular exercise routines, and resistance and strength routines (both basic and advanced levels).

The intervention was designed as self-access, asynchronous education and was delivered as an adjunct to usual HIV clinical care, which consisted of routine outpatient HIV follow-up, including clinical review, antiretroviral treatment monitoring, laboratory assessment, management of comorbidities, and concomitant medication, and general lifestyle counseling when clinically indicated. No structured exercise or dietary program was routinely provided outside the study intervention. No log-in credentials were required. At the baseline visit, the investigator demonstrated how to access the videos through the QR codes using the participants’ own mobile devices. During follow-up, no structured technical support, reminders, or reinforcement of video use was provided beyond informal assistance that participants could receive from relatives or caregivers. The videos were publicly accessible through the QR links, the links remained stable throughout the study period, no registration was required, and the QR-linked materials did not include advertisements or unrelated recommendations.

### Outcomes

#### Primary Outcome

The primary outcome was change in frailty phenotype category (robust, prefrail, or frail) at 6 months and transitions between categories. Frailty was assessed according to the Fried frailty phenotype. Participants were classified as robust (0 criteria), prefrail (1-2 criteria), or frail (≥3 criteria). The five phenotype components were evaluated as follows: (1) unintentional weight loss, defined as a loss of 5 kg or more or 5% or more of body weight during the previous year; (2) exhaustion, defined as self-report of feeling that everything was an effort or being unable to get going for at least 3 days during the previous week; (3) weakness, assessed via grip strength measured in the dominant hand using a hand dynamometer, applying sex- and BMI-specific cutoffs; (4) slowness, assessed through a timed 10-m walk at usual pace, with slow gait defined according to height-specific thresholds (≥15.3 seconds for participants measuring ≤173 cm and ≥13.1 seconds for participants measuring >173 cm); and (5) low physical activity, defined as weekly energy expenditure of less than 383 kcal/week in men and less than 270 kcal/week in women.

#### Secondary Outcomes

Secondary outcomes included changes in individual Fried frailty criteria (weight loss, exhaustion, grip strength, gait speed, and low physical activity) and patient-reported outcomes (Insomnia Severity Index [ISI]; 10-item Connor-Davidson Resilience Scale [CD-RISC-10]; University of California, Los Angeles, Loneliness Scale [ULS]; and Hospital Anxiety and Depression Scale [HADS] anxiety and depression categories). Lifestyle measures included physical activity, assessed using the International Physical Activity Questionnaire (IPAQ), and adherence to the Mediterranean diet, assessed using the 14-item Mediterranean Diet Adherence Screener (MEDAS) developed for the PREDIMED study [11]. The MEDAS score ranges from 0 to 14 points, with higher scores indicating greater adherence to the Mediterranean diet. Each item is scored dichotomously (0 or 1), and the total score is obtained by summing all item scores. Laboratory and immunologic markers included high-sensitivity C-reactive protein (hsCRP), interleukin-6 (IL-6), D-dimer, and the CD4-to-CD8 ratio. HADS outcomes were analyzed categorically according to established anxiety and depression classification thresholds.

### Statistical Analysis

We summarized baseline and 6-month outcomes using appropriate descriptive statistics (medians and IQRs or means and SDs depending on distribution; categorical variables were reported as numbers and percentages). Frailty transitions were summarized using a transition matrix. Given the small sample size and sparse cells across the paired 3-category frailty outcome, analyses of frailty transitions were considered primarily descriptive rather than based on formal global inferential testing. The McNemar test was used only for paired dichotomous categorical variables. For paired continuous outcomes, comparisons were performed using the paired Student *t* test or, when normality assumptions were not met, the Wilcoxon signed-rank test. Because several psychosocial outcomes were nonnormally distributed, these variables are reported as medians and IQRs, and standardized effect sizes were calculated as *r*=*Z*/√*N* to complement *P* values and provide an estimate of the magnitude of change independent of sample size. Given the exploratory design and limited sample size, analyses of secondary outcomes were not adjusted for multiple comparisons and should therefore be interpreted as hypothesis generating. All statistical tests were 2-tailed. Statistical significance was set at a *P* value below .05. Analyses were performed using SPSS (version 28; IBM Corp).

### Reporting Guideline

This quasi-experimental, uncontrolled before-and-after study was reported following the TREND (Transparent Reporting of Evaluations with Nonrandomized Designs) statement (Multimedia Appendix 2), and the intervention was described using the TIDieR (Template for Intervention Description and Replication) checklist. The QR code–enabled video resources are provided in Multimedia Appendix 3 to ensure reproducibility [12,13].

## Results

### Participant Flow and Baseline Context

The study enrolled 52 participants at baseline; the cohort was predominantly male. At baseline, 7.7% (n=4) were classified as frail, 50% (n=26) were classified as prefrail, and 42.3% (n=22) were classified as robust. For the 6-month analysis, follow-up data were available for 96.2% (n=50) of the participants (available-case analysis). The 3.8% (n=2) of the participants without 6-month follow-up data declined to continue in the study; at baseline, one had been classified as prefrail and the other as robust.

### Primary Outcome: Frailty Transitions at 6 Months

At 6 months, the overall distribution remained broadly similar (robust: 16/50, 32%; prefrail: 26/50, 52%; frail: 8/50, 16%). Frailty transitions were frequent and bidirectional and, in this sample, occurred only between adjacent states; given the small sample size and sparse cells, these analyses were interpreted descriptively (Table 1). Of the 21 participants who were robust at baseline, 11 (52.4%) remained robust, and 10 (47.6%) transitioned to prefrail. Of the 25 participants classified as prefrail at baseline, 5 (20%) improved to robust, 14 (56%) remained prefrail, and 6 (24%) progressed to frail. Of the 4 participants classified as frail at baseline, 2 transitioned to prefrail, and 2 remained frail. No direct transitions between the robust and frail categories were observed.

**Table 1:** Transition matrix of frailty phenotype status from baseline to 6 months in older people living with HIV participating in a pragmatic single-arm pilot implementation study of a QR code–enabled lifestyle education intervention conducted in an HIV outpatient clinic in southern Spain.

	Robust at month 6 (n=16), n (%)	Prefrail at month 6 (n=26), n (%)	Frail at month 6 (n=8), n (%)
Robust at baseline (n=21)	11 (52.4)	10 (47.6)	0 (0)
Prefrail at baseline (n=25)	5 (20)	14 (56)	6 (24)
Frail at baseline (n=4)	0 (0)	2 (50)	2 (50)

### Secondary Outcomes

#### Changes in Frailty Criteria

Across the individual Fried frailty criteria, we did not observe statistically significant changes in the proportion of participants meeting each criterion over the 6-month period. Notably, unintentional weight loss showed a favorable trend, with 5 of 6 participants who reported weight loss at baseline no longer reporting it at follow-up. In contrast, grip strength did not improve among participants with impaired baseline strength, suggesting limited impact on objective strength over the follow-up period.

#### Lifestyle Measures

Changes in continuous secondary outcomes were generally modest over the 6-month follow-up period. Mediterranean diet adherence remained stable (median score 11.0, IQR 9-13 at both time points; *P*=.19). Median physical activity scores showed a small nonsignificant decrease from 12.0 (IQR 10-14) at baseline to 11.0 (IQR 9-13) at 6 months (*P*=.14). These findings suggest limited changes in lifestyle-related measures over the follow-up period.

#### Inflammatory and Immunologic Markers

Similarly, inflammatory and immunologic markers did not show statistically significant changes over 6 months, including hsCRP, IL-6, D-dimer, and the CD4-to-CD8 ratio (Table 2).

**Table 2:** Changes in inflammatory and immunologic biomarkers from baseline to 6 months in older people living with HIV enrolled in a pragmatic single-arm pilot implementation study of a QR code–enabled self-access lifestyle education intervention in southern Spain.

Parameter	Baseline, median (IQR)	Month 6, median (IQR)	*P* value
D-dimer (ng/mL; n=41)	264 (200-320)	308 (240-360)	.053
hsCRP^a^ (mg/L; n=49)	3.0 (2.0-4.5)	3.2 (2.1-4.6)	.90
IL-6^b^ (pg/mL; n=40)	2.75 (2.0-4.0)	2.45 (1.8-3.8)	.14
CD4-to-CD8 T-cell ratio (n=50)	0.99 (0.88-1.10)	1.01 (0.89-1.12)	.81

^a^hsCRP: high-sensitivity C-reactive protein.

^b^IL-6: interleukin-6.

#### Psychosocial Outcomes

Psychosocial measures showed a mixed pattern. Insomnia severity improved, with the ISI median score decreasing from 6.5 (IQR 4-9) at baseline to 4.0 (IQR 2-7) at 6 months (*P*=.001; *r*=0.49). Resilience increased, with the CD-RISC-10 median score increasing from 32.0 (IQR 28-36) to 37.0 (IQR 33-40; *P*<.001; *r*=0.65). In contrast, perceived loneliness worsened, with the ULS median score increasing from 34.5 (IQR 30-39) to 38.0 (IQR 34-42; *P*=.001; *r*=0.49).

HADS outcomes were analyzed categorically. No significant changes were observed in anxiety or depression categories over the 6-month follow-up period. For depression, 10% (5/50) of the participants showed a favorable category change, 6% (3/50) worsened, and 84% (42/50) remained unchanged (*P*=.36). For anxiety, 22% (11/50) of the participants showed a favorable category change, 6% (3/50) worsened, and 72% (36/50) remained unchanged (*P*=.15).

## Discussion

### Principal Findings

In this prospective, single-arm evaluation of a QR code–enabled, self-access lifestyle education program delivered to older people living with HIV, we observed three main findings over 6 months: (1) frailty status was highly dynamic, with frequent bidirectional transitions between adjacent states and no direct robust-frail transitions in this sample; (2) insomnia severity and resilience scores changed in a favorable direction, whereas loneliness scores worsened; and (3) there were no clear short-term changes in physical activity, Mediterranean diet adherence, grip strength, or inflammatory and immunologic markers. Given the uncontrolled design, these findings should be interpreted as observed associations during follow-up rather than as effects attributable to the intervention.

Baseline context is important for interpreting these transitions. In our previously published cross-sectional analysis of the same cohort (N=52; median age 64 years), frailty prevalence based on the Fried phenotype was 7.7% (4/52), prefrailty prevalence was 50% (26/52), and robustness prevalence was 42.3% (22/52) [14].

The observed pattern of adjacent-state transitions aligns with cohort evidence in older people living with HIV showing that frailty is not static and often fluctuates over time, with transitions concentrated between neighboring states rather than occurring in abrupt jumps [3,4].

### Interpreting the Limited Physical and Biological Change

Despite providing structured education on exercise and diet, we did not detect improvements in physical activity measures or grip strength. This is consistent with broader evidence that unsupervised or minimally supported home-based exercise interventions can be limited by variable adherence and that measurable improvements in strength often require sufficient training intensity, progression, and engagement—features that may be difficult to achieve through self-access education alone. Systematic reviews indicate that digital supports can improve adherence to home exercise in some contexts, but effects vary and may diminish over time without additional reinforcement [15-21].

Similarly, no statistically significant changes were observed in inflammatory or immunologic markers over 6 months. Although D-dimer and IL-6 showed divergent numerical trends, these findings should be interpreted cautiously given the small sample size, lack of statistical significance, and exploratory design. Longer follow-up and more intensive, monitored interventions may be needed to determine whether lifestyle-focused digital strategies can influence inflammatory or coagulation pathways in older people living with HIV [22-30].

### Role of QR Codes and Self-Access Digital Education: Plausible Mechanisms and Limitations

A distinctive aspect of our program was the QR code bridge from a paper leaflet to self-access YouTube education. QR-based approaches are attractive because they are low cost and scalable and reduce friction (no app installation). Prior work suggests that QR code interventions in health care are generally feasible and well accepted, but challenges include smartphone availability, connectivity, and user confidence [9,10].

However, adoption of health technologies in older adults with chronic diseases is influenced by multiple barriers (digital literacy, perceived usefulness, accessibility, physical limitations, privacy concerns, and motivational factors). These barriers may be particularly relevant in frail or multimorbid individuals and can reduce sustained engagement with self-access resources [6].

The QR-linked YouTube videos were publicly accessible, did not require registration, and were curated by hospital departments to reduce content quality concerns (Multimedia Appendix 3). The links remained stable during the study period, and the materials did not include advertisements or unrelated recommendations; nevertheless, future studies should prospectively monitor link stability, access barriers, and platform-related usability issues [31].

### Why Insomnia and Resilience Improved Whereas Loneliness Worsened

In this context, our prior pilot study of a privacy-preserving mobile app that enabled anonymous peer-to-peer contact (GoSHAPE) suggested that confidential digital peer support may help mitigate loneliness in older people living with HIV, complementing lifestyle education and potentially addressing a key social vulnerability that our QR code–enabled self-access program did not improve [31].

The observed changes in insomnia and resilience may reflect several non–mutually exclusive mechanisms, including increased self-efficacy from structured guidance, sleep-related behavior changes, or nonspecific contextual effects such as increased perceived support from receiving tailored resources. The observed worsening in perceived loneliness warrants careful interpretation [7]. Given the uncontrolled design and the absence of intervention exposure data, we cannot determine whether this finding reflects temporal psychosocial variation, broader contextual influences, or the limitations of a self-access educational strategy that did not actively target social connectedness. These possibilities should be considered hypotheses for future study rather than explanations directly supported by the present data. This is clinically important given the recognized association between both loneliness and social vulnerability and frailty risk in older people living with HIV [5].

### Implications for Practice and Future Program Design

These exploratory findings support a blended approach in future studies: scalable QR code–enabled education should be combined with components that target (1) engagement and adherence (eg, QR scan tracking, video analytics, scheduled reminders, participant feedback, motivational interviewing, or progress tracking), (2) progressive resistance training with supervision or structured progression, and (3) social prescribing or group-based interventions to counter loneliness. There is emerging evidence suggesting that digitally delivered group-based or supervised exercise may improve feasibility and outcomes in older adults.

### Limitations

This study has several important limitations. First, the single-arm uncontrolled design precludes causal interpretation; observed changes may reflect natural fluctuation in frailty status, regression to the mean, concurrent routine care, or other unmeasured contextual factors. Second, this study was underpowered to detect clinically meaningful changes in frailty categories, grip strength, and inflammatory and immunologic markers, particularly given the small number of participants in some baseline subgroups (eg, participants classified as frail). Third, multiple secondary outcomes were analyzed without adjustment for multiple comparisons; therefore, statistically significant findings, particularly for psychosocial outcomes, should be interpreted cautiously and as hypothesis generating. Fourth, no objective or structured self-reported measures of intervention exposure were collected (eg, QR scans, video views, watch time, or adherence), and no structured technical support or reinforcement was provided after the baseline demonstration. This makes it impossible to distinguish between limited intervention efficacy and limited participant engagement with the materials. Fifth, the single-center setting; predominantly male sample; and exclusion of individuals with greater dependency, dementia, severe mobility limitations, or serious comorbidities limit generalizability. Sixth, reliance on self-reported lifestyle outcomes may have introduced measurement bias.

### Conclusions

In this pragmatic single-arm exploratory study, selected psychosocial outcomes changed over 6 months following delivery of a QR code–enabled, self-access lifestyle education strategy, whereas no clear short-term changes were observed in physical activity, grip strength, diet adherence, or inflammatory and immunologic markers. Frailty remained highly dynamic, with frequent bidirectional transitions. Given the lack of a control group, small sample size, unadjusted multiple comparisons, and absence of intervention exposure metrics, these findings should be interpreted as exploratory and hypothesis generating rather than as evidence of causal effect. Future multimodal studies should combine scalable digital education with strategies to enhance engagement, quantify intervention use through QR tracking and video analytics, incorporate reminders and participant feedback, support progressive resistance training uptake, and promote social connectedness.

## Data Availability

The data that support the findings of this study are available on request from the corresponding author.

## Appendix

Multimedia Appendix 1 Trifold leaflet used in the QR code–enabled self-access lifestyle education intervention (HIDRA 360), including links to Mediterranean diet and home-based aerobic and resistance exercise videos for older people living with HIV in southern Spain.

Multimedia Appendix 2 TREND (Transparent Reporting of Evaluations with Nonrandomized Designs) checklist for a pragmatic single-arm pilot implementation study using a quasi-experimental pretest-posttest design to evaluate a QR code–enabled self-access lifestyle education intervention in older people living with HIV.

Multimedia Appendix 3 Curated YouTube videos linked through QR codes in the self-access lifestyle education intervention, including nutrition, physical activity information, and home exercise routines (basic and advanced levels) for older people living with HIV.

## Abbreviations

CD-RISC-10: 10-item Connor-Davidson Resilience Scale
HADS: Hospital Anxiety and Depression Scale
hsCRP: high-sensitivity C-reactive protein
IL-6: interleukin-6
IPAQ: International Physical Activity Questionnaire
ISI: Insomnia Severity Index
MEDAS: Mediterranean Diet Adherence Screener
TIDieR: Template for Intervention Description and Replication
TREND: Transparent Reporting of Evaluations with Nonrandomized Designs
ULS: University of California, Los Angeles, Loneliness Scale
