# Supplementary material for: QR Code–Enabled Self-Access Lifestyle Education in Older People Living With HIV: Pragmatic Pilot Quasi-Experimental Pretest-Posttest Implementation Study
Source: J Med Internet Res. 2026 Jul 29;28:e93633. doi: 10.2196/93633 (PMC13469835; doi:10.2196/93633)

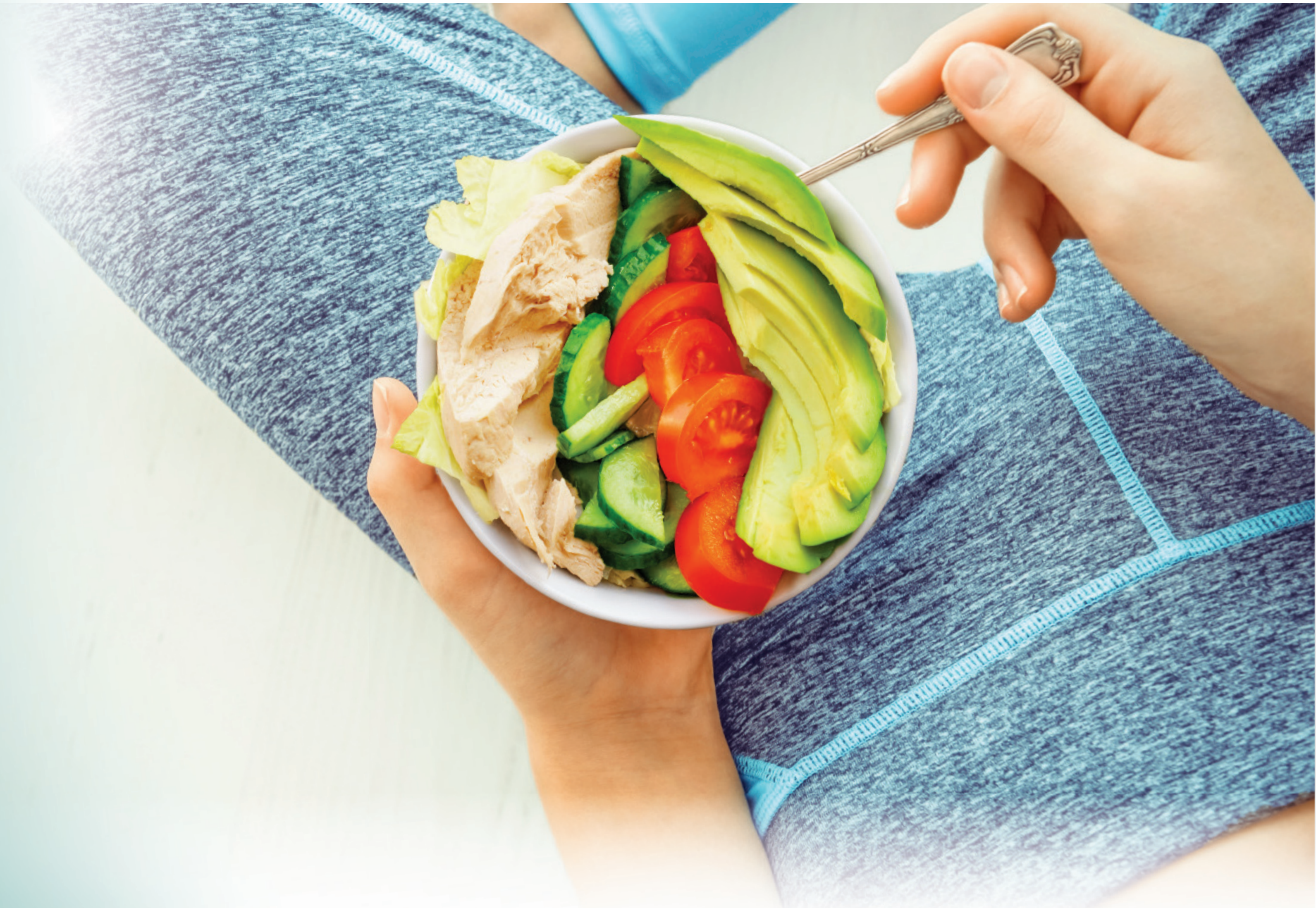

PROGRAMA
DE ENVEJECIMIENTO SALUDABLE
HIDRA 360

Para cualquier duda acerca del programa de actividad física, puede contactar con nuestro equipo en el correo electrónico:

pedroj.alarcon.f.sspa@juntadeandalucia.es

SERVICIO ANDALUZ DE SALUD
Consejería de Salud y Consumo

Área Hospitalaria
Costa del Sol

w w w . h c s . e s

PROGRAMA DE ENVEJECIMIENTO SALUDABLE

El programa Hidra 360 tiene como objetivo promover en los participantes hábitos de vida saludables en relación con la actividad física y la alimentación. Usted recibe esta hoja informativa porque ha decidido formar parte del programa. Todo el equipo de personal sanitario que formamos parte del proyecto le agradecemos su participación.

En este documento queremos proporcionarle acceso a información importante acerca de nutrición saludable y actividad física, así como a un programa de ejercicio físico adaptado a sus condiciones y diseñado para que pueda realizarlo en su propio domicilio.

Todos estos contenidos están disponibles en formato vídeo en el canal de YouTube del Área Hospitalaria Costa del Sol. Para facilitarle el acceso a los mismos, a continuación le indicaremos la dirección URL y un código QR de cada vídeo. Sólo tiene que teclear la dirección que le indicamos en su navegador de internet,

INFORMACIÓN SOBRE NUTRICIÓN SALUDABLE Y DIETA MEDITERRÁNEA

o escanear el código con un lector de QR para acceder al vídeo correspondiente.

En este vídeo se le proporcionan nociones generales sobre nutrición, información acerca de los beneficios de la dieta mediterránea y propuestas concretas sobre cómo mejorar su forma de alimentarse aplicándola.

INFORMACIÓN SOBRE ACTIVIDAD FÍSICA

- 10 RAZONES PARA EMPEZAR UN PROGRAMA DE ENTRENAMIENTO

En este vídeo queremos informarle de los beneficios científicamente demostrados que puede obtener al realizar un programa de ejercicio físico.

- RECOMENDACIONES GENERALES

En este vídeo le proporcionamos unas directrices generales en relación con el programa de ejercicio físico que le proponemos.

- ENTRENAMIENTO CARDIOVASCULAR

En este vídeo se le proporcionan recomendaciones y propuestas para llevar a cabo la parte de entrenamiento cardiovascular del programa.

- ENTRENAMIENTO DE FUERZA

En este vídeo le indicamos le explicamos en qué consiste el programa de ejercicio domiciliario que le proponemos, de qué partes consta, cómo llevarlo a cabo y cómo progresar para obtener el máximo beneficio del entrenamiento.

PROGRAMA DE EJERCICIO FÍSICO EN DOMICILIO

Esta última parte constituye el programa de ejercicio que le proponemos realizar en su domicilio. Consta de tres días a la semana, y está dividido en dos niveles de dificultad en relación con la condición física de la persona que lo realiza.

Si nunca ha realizado un programa de ejercicio, o ha pasado mucho tiempo desde la última vez que realizó un entrenamiento le proponemos que empiece con la **RUTINA NIVEL BÁSICO:**

- DÍA 1
- DÍA 2
- DÍA 3

Si realiza ejercicio físico de forma regular en la actualidad, puede intentar empezar con la **RUTINA NIVEL AVANZADO:**

- DÍA 1
- DÍA 2
- DÍA 3

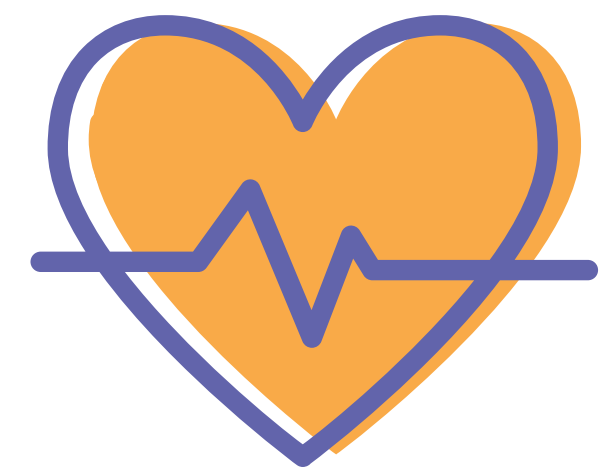

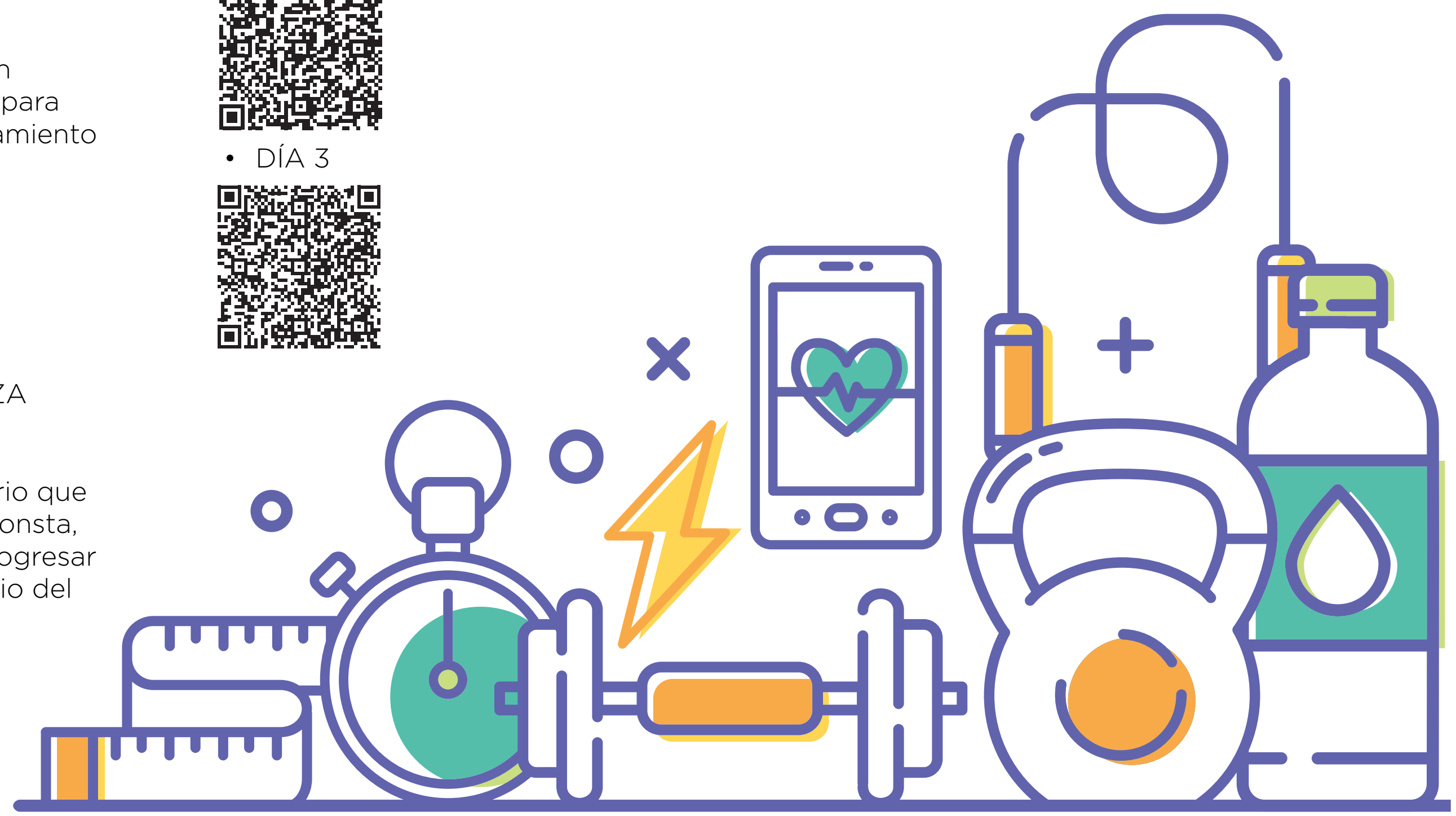

Supplement: Supplementary file 1 [file jmir_v28i1e93633_app1.pdf]
