# Supplementary material for: QR Code–Enabled Self-Access Lifestyle Education in Older People Living With HIV: Pragmatic Pilot Quasi-Experimental Pretest-Posttest Implementation Study
Source: J Med Internet Res. 2026 Jul 29;28:e93633. doi: 10.2196/93633 (PMC13469835; doi:10.2196/93633)
Supplement: Supplementary file 2 [file jmir_v28i1e93633_app2.pdf]

# Multimedia Appendix 2. QR code–linked videos used in the intervention (title and URL)

This appendix lists the videos accessible via the QR codes provided in the intervention leaflet (Hydra 360 program). Titles reflect the leaflet headings; URLs correspond to the decoded QR links.

## Nutrition

| Video | URL |
|---|---|
| Healthy nutrition and Mediterranean diet | https://www.youtube.com/watch?v=xXJnaC84aEU&list;=PL71_whP-7dEn6Ze Xbmf-at83qOXbMumZv&index;=2&ab;_channel=HospitalCostadelSol |

## Physical activity — Information videos

| Video | URL |
|---|---|
| 10 reasons to start an exercise program | https://www.youtube.com/watch?v=BOgiypmPDVg&list;=PL71_whP-7dEn6Ze Xbmf-at83qOXbMumZv&index;=2 |
| General recommendations | https://www.youtube.com/watch?v=xXJnaC84aEU&list;=PL71_whP-7dEn6Ze Xbmf-at83qOXbMumZv&index;=3&ab;_channel=HospitalCostadelSol |
| Cardiovascular training | https://www.youtube.com/watch?v=ule7kqoBKBw&list;=PL71_whP-7dEn6Ze Xbmf-at83qOXbMumZv&index;=4&ab;_channel=HospitalCostadelSol |
| Strength training | https://www.youtube.com/watch?v=iKYRL-0uVrA&list;=PL71_whP-7dEn6Ze Xbmf-at83qOXbMumZv&index;=5&ab;_channel=HospitalCostadelSol |

## Physical activity — Home exercise program (Basic level)

| Routine | URL |
|---|---|
| Day 1 | https://www.youtube.com/watch?v=RBelDj6xRPc&list;=PL71_whP-7dEn6Ze Xbmf-at83qOXbMumZv&index;=6&ab;_channel=HospitalCostadelSol |
| Day 2 | https://www.youtube.com/watch?v=FWgPkOKiSG8&list;=PL71_whP-7dEn6Ze Xbmf-at83qOXbMumZv&index;=8&ab;_channel=HospitalCostadelSol |
| Day 3 | https://www.youtube.com/watch?v=-JmPNI2kVOM&list;=PL71_whP-7dEn6Ze Xbmf-at83qOXbMumZv&index;=10&ab;_channel=HospitalCostadelSol |

## Physical activity — Home exercise program (Advanced level)

| Routine | URL |
|---|---|
| Day 1 | https://www.youtube.com/watch?v=rnNU4_rustg&list;=PL71_whP-7dEn6Ze Xbmf-at83qOXbMumZv&index;=9&ab;_channel=HospitalCostadelSol |
| Day 2 | https://www.youtube.com/watch?v=AJdoZZgXa6U&list;=PL71_whP-7dEn6Ze Xbmf-at83qOXbMumZv&index;=7&ab;_channel=HospitalCostadelSol |
| Day 3 | https://www.youtube.com/watch?v=Iik97vGRkNQ&list;=PL71_whP-7dEn6Ze Xbmf-at83qOXbMumZv&index;=11&ab;_channel=HospitalCostadelSol |
