# Supplementary material for: QR Code–Enabled Self-Access Lifestyle Education in Older People Living With HIV: Pragmatic Pilot Quasi-Experimental Pretest-Posttest Implementation Study
Source: J Med Internet Res. 2026 Jul 29;28:e93633. doi: 10.2196/93633 (PMC13469835; doi:10.2196/93633)
Supplement: Supplementary file 3 [file jmir_v28i1e93633_app3.pdf]

Multimedia Appendix 3. TREND Checklist.

Manuscript: "QR code-enabled self-access lifestyle education in older people living with HIV: a pragmatic pilot implementation study."

Item	Section/Topic	Requirement (summary)	Reported?	Where reported in manuscript (section)
1	Title and Abstract	Information on allocation/assignment and target population; structured abstract.	Yes	Title; Abstract (Background/Objective/Methods/Results/Conclusions). Note: allocation not applicable (single-group).
2	Introduction-Background	Scientific background and rationale.	Yes	Introduction (paras 1-6).
3	Methods-Participants	Eligibility criteria; recruitment method and setting; data collection setting.	Yes	Methods > Participants; Study design and setting; Results > Participant flow and baseline context.
4	Methods-Interventions	Intervention content, delivery, provider, setting, dose/duration, and adherence support.	Yes	Methods > Intervention; Multimedia Appendices 1-2 (leaflet and QR-linked video list).
5	Methods-Objectives	Specific objectives/hypotheses.	Yes	Objective section; Methods > Outcomes.
6	Methods-Outcomes	Primary/secondary outcomes; measurement methods; validated instruments.	Yes	Methods > Outcomes; instruments listed (FF, ISI, CD-RISC-10, UCLA, HADS, IPAQ, Andalusian Mediterranean diet questionnaire).
7	Methods-Sample size	How sample size was determined; interim analyses/stopping rules if any.	No	Not stated. Pragmatic sample (all eligible invited) / feasibility evaluation; no formal power calculation.
8	Methods-Assignment method	Unit of assignment; method of assignment; steps to minimize bias due to nonrandomization.	NA	Uncontrolled before-after design (no allocation to study arms). Unit is individual; pre-post within-person comparison.
9	Methods-Blinding	Whether blinding was used and how assessed.	NA	No blinding described/typically not applicable for this design.
10	Methods-Unit of analysis	Smallest unit analyzed; methods for correlated data if applicable.	Yes	Methods > Statistical analysis (paired tests; within-person comparisons).
11	Methods-Statistical methods	Statistical methods for primary/secondary outcomes; handling missing data; software.	Yes	Methods > Statistical analysis (descriptive summaries, paired tests, P threshold, SPSS v28). Missing-data approach stated as available-case in Results.
12	Results-Participant flow	Enrollment, follow-up, analysis; numbers at each stage; deviations; flow diagram recommended.	Partly	Results > Participant flow and baseline context (baseline n=52; follow-up n=50). Reasons for missing follow-up reported; flow diagram not included.
13	Results-Recruitment	Dates defining recruitment and follow-up periods.	No	Recruitment and follow-up dates not reported.
14	Results-Baseline data	Baseline demographic/clinical characteristics; comparison of retained vs lost if relevant.	Partly	Results > Participant flow and baseline context (age/sex summary, baseline frailty distribution, and baseline frailty status of participants lost to follow-up). Full baseline characteristics table not included in this follow-up paper.

Item	Section/Topic	Requirement (summary)	Reported?	Where reported in manuscript (section)
15	Results-Baseline equivalence	Group equivalence at baseline and methods to adjust (if multiple groups).	NA	Single-group study (baseline equivalence across groups not applicable).
16	Results-Numbers analyzed	Denominators for each analysis; approach to noncompliers; ITT vs available-case.	Yes	Results (available-case n=50) and denominators reported in transition matrix (Table 1) and outcomes.
17	Results-Outcomes and estimation	Results for each outcome; effect size/CI where applicable; null findings.	Partly	Results (transitions; medians [IQR]; P values; null findings). Standardized effect sizes are reported for psychosocial outcomes, but confidence intervals for paired changes are not reported.
18	Results-Ancillary analyses	Other analyses (subgroups, adjusted); pre-specified vs exploratory.	NA	No ancillary/subgroup analyses reported.
19	Results-Adverse events	Important harms/unintended effects.	No	Adverse events not reported (none observed/reported).
20	Discussion-Interpretation	Interpretation with limitations/bias; mechanisms; implementation barriers; implications.	Yes	Discussion (Principal findings; Interpretation of limited physical/biological change; QR limitations; loneliness; implications; limitations).
21	Discussion-Generalizability	External validity given population, setting, follow-up, adherence, context.	Yes	Discussion > Limitations and general discussion of scalability/setting.
22	Discussion-Overall evidence	Interpretation in context of existing evidence and theory.	Yes	Discussion integrates findings with prior evidence (citations) and theory/interpretation.

Notes: “NA” indicates not applicable to a single-group uncontrolled before-after design. “Partly” indicates partial reporting; see comments in the “Where reported” column.

Source checklist: Des Jarlais DC, Lyles C, Crepaz N; TREND Group. Am J Public Health. 2004;94(3):361-366.
